# Association of Urine (pH < 5.5) with Community Periodontal Index (CPI) and the Number of Remaining Teeth in Korean Adults: A Cross-Sectional Study Using Data from Korea National Health and Nutrition Examination Survey 2016–2018

**DOI:** 10.3390/healthcare12070740

**Published:** 2024-03-29

**Authors:** Su-Yeon Hwang, Jung-Eun Park

**Affiliations:** 1 Department of Dental Hygiene, Daejeon Institute of Science and Technology, Daejeon 35408, Republic of Korea; hsyen@dst.ac.kr; 2 Department of Dental Hygiene, College of Health Science, Dankook University, Cheonan 31116, Republic of Korea

**Keywords:** Community Periodontal Index (CPI), Korea National Health and Nutrition Examination Survey (KNHANES), oral health, urine

## Abstract

This study aims to determine the association between UpH (<5.5), Community Periodontal Index (CPI), and the number of remaining teeth—cumulative indicators of oral health—using data from the 7^th^ Korea National Health and Nutrition Examination Survey (KNHANES, 2016–2018), which represents the Korean population. Data from 12,689 adults aged 19 years and older who had periodontal examinations were analyzed. Logistic regression analysis was performed after adjusting for demographic, health, and health-related behavioral factors as covariates to determine the association between UpH, CPI, and the number of remaining teeth. This study found that UpH (<5.5) was associated with CPI and the number of remaining teeth. For UpH (<5.5), the odds ratio for CPI (≥4 mm) was 1.19 times (95% CI: 1.06–1.33). The risk of tooth loss was 1.25 times (95% CI: 1.06–1.48) for those with 0–19 remaining teeth and 1.20 times (95% CI: 1.07–1.34) for those with 20–27 teeth. The results revealed an association between UpH, CPI, and the number of remaining teeth. However, further longitudinal research on UpH and oral status is necessary.

## 1. Introduction

Urine pH (UpH) refers to urine acidity. On average, UpH is slightly acidic with a pH of 6.0, and acidic urine means a pH of less than 5.5 [1]. It is known to be influenced by systemic factors such as an individual’s diet, insulin resistance, hydration status, diuretics, and chronic kidney disease medications [2,3,4,5].

Previous studies have reported that UpH (<5.5) is associated with metabolic syndrome (Mets), obesity, diabetes development, and insulin resistance [6,7,8]. In addition, a retrospective cohort study of predictors of acidic urine and Mets has found that acidic urine is associated with a higher risk of developing Mets [9]. These results suggest that insulin resistance may be involved in the reduction in ammonium excretion in the proximal tubule, which acts as a buffer for urine [6].

Additionally, previous studies with gout patients have suggested that insulin resistance plays an important role in acidic urine [10], and longitudinal analysis has indicated that it is a risk factor for the new onset of Mets within 5 years among participants with acidic urine [9]. Therefore, acidic urine seems to be influenced by the participant’s systemic condition related to insulin resistance and a healthy lifestyle. Research on metabolic disorders and insulin resistance using UpH has been conducted. However, there is limited research on the oral cavity and other organs using UpH.

Previous studies related to the oral cavity have reported that insulin resistance is associated with an increased risk of inflammation of the gingival and periodontal tissues in the oral cavity. Alternatively, it has been suggested that the presence of periodontal tissue inflammation may reflect undetected insulin resistance [11].

A previous study reported that tooth loss is considered a cumulative indicator of lifelong oral health among the indicators of oral health [12]. Among the various bacteria that contribute to periodontitis, virulence factors of *Porphyromonas gingivalis* have been suggested to be the main cause of tooth loss [13]. Through multiple virulence factors, the bacterium interferes with the host’s immune system, triggers an inflammatory response, and induces insulin resistance [14]. Notably, oral inflammation is correlated with systemic diseases, such as diabetes, non-alcoholic fatty liver disease, and cardiovascular diseases [15].

According to these findings, insulin resistance might lead to acidic urine induction and periodontal tissue inflammation. However, research on the association between UpH (<5.5) and oral disease is scarce. Therefore, understanding the association between Community Periodontal Index (CPI) and tooth loss according to the level of UpH will contribute to the study of oral systemic conditions and biological markers.

This study was conducted based on the research hypothesis that UpH (<5.5) would be associated with CPI and tooth loss, which are indicators of oral health.

Therefore, this study aims to determine the association between UpH (<5.5), CPI, and the number of remaining teeth using data from the 7^th^ Korea National Health and Nutrition Examination Survey (KNHANES, 2016–2018), which represents the Korean population.

## 2. Materials and Methods

### 2.1. Study Participants

This study utilized the 7^th^ KNHANES, a nationally representative cross-sectional survey conducted from 2016 to 2018. Year 1 and 2 (2016–2017) data of the 7^th^ KNHANES were collected without an Institutional Review Board (IRB) review in accordance with the Bioethics and Safety Act, and year 3 (2018) data were collected with approval from the IRB of the Korea Disease Control and Prevention Agency (IRB No. 2018-01-03-P-A). Out of a total of 16,489 individuals, 13,199 individuals who were over the age of 19 were analyzed. Out of a total of 16,489 participants who took part in the health survey and screening examination, 13,199 participants aged 19 years and older were extracted. Among them, 12,689 participants who underwent periodontal tissue examination were selected for the analysis. In the results, the discrepancy in the total frequency is due to missing data. This study utilized data from the 7^th^ Korea National Health and Nutrition Examination Survey, and the results of oral examinations were analyzed.

### 2.2. Number of Teeth

The measurement of the dependent variable, tooth count, was conducted by highly trained public health dentists using standardized methods. The training was provided through orthodontic lectures, training using tooth models, web-based photo and human oral examination simulations, and on-site training to ensure the reliability of the oral examination. The number of remaining teeth was calculated after excluding the third molars. The number of remaining teeth of the participants was categorized into three groups: ≤19, 20–27, and 28 teeth, according to the classification of previous studies [16].

### 2.3. Community Periodontal Index (CPI)

Periodontal examinations for CPI, a dependent variable, were performed by highly trained public health dentists with ongoing quality control. The examinations were conducted according to the CPI screening criteria set forth by the World Health Organization (WHO) [17]. Periodontal pocket depths were measured at six sites (maxillary right posterior, maxillary anterior, maxillary left posterior, mandibular right posterior, mandibular anterior, and mandibular left posterior). Periodontal pocket depth was measured using the WHO probe, with markings at 3.5 mm and 5.5 mm. The CPI criteria were as follows: 0 (healthy and no signs of inflammation), 1 (gingival bleeding), 2 (calculus), 3 (shallow pocket; >3.5 mm and ≤5.5 mm), and 4 (deep pocket; >5.5 mm). For the CPI record, index teeth or teeth remaining in the sextant were evaluated, and the highest score was recorded. In this study, we defined a pocket depth of <4 mm (CPI 0–2) and ≥4 mm (CPI 3, 4).

### 2.4. Urine Ph

Urine samples were collected in the morning after fasting overnight. UpH was measured using a Urisys 2400 analyzer (Roche, Mannheim, Germany). The measured values were categorized into 5.0, 5.5, 6.0, 6.5, 7.0, 7.5, 8.0, 8.5, and 9.0. Based on previous studies, the samples were classified as <5.5 and ≥5.5 [18,19].

### 2.5. Covariates

Each variable was reclassified as follows. Demographic factors included gender, age, household income level, and education. Age was categorized into 19–29, 30–39, 40–49, 50–59, and 60+. Household income quartiles were categorized into three groups: high, middle (mid-high and mid-low), and low. Education level was categorized into less than an elementary school diploma, middle school diploma, high school diploma, and college degree or higher.

For health factors, having diabetes means a fasting blood glucose of ≥126 mg/dL, diagnosis by a physician, taking glycemic control medication, or injecting insulin [20]. The body mass index (BMI) was categorized into underweight (<18.5 kg/m^2^), normal weight (18.5–22.9 kg/m^2^), overweight (23–24.9 kg/m^2^), and obese (>25 kg/m^2^).

For health-related behavioral factors, current smoking status was divided into non-smoking (smoking less than five packs) and smoking (smoking more than five packs) [21,22]. Drinking status was divided into non-drinking (lifetime non-drinking or drinking less than one drink per month in the past year) and drinking (drinking more than one drink per month in the past year) [23]. Daily toothbrushing frequency was divided into ≤ 1 times/day, and ≥2 times/day.

### 2.6. Statistical Analysis

The KNHANES data were collected using a complex sample design, and a composite sample analysis was conducted after applying weights, stratification variables (kstrata), and primary sampling unit (psu).

A cross-analysis was conducted on UpH, CPI, and the number of remaining teeth according to the general characteristics of the participants. The association between UpH and the number of remaining teeth was assessed using the Multinomial logistic regression models. The association between UpH and CPI was evaluated using logistic regression models.

Model 1 is the unadjusted model, and Model 2 is the adjusted model for gender, age, household income, education, diabetes, BMI, smoking, drinking, and daily toothbrushing frequency. Results are presented as odds ratios for tooth loss and CPI, with 95% confidence intervals (CIs).

All statistical analyses were conducted using the SPSS Windows software version 20.0, and significance was tested at a Type I error level of 0.05.

## 3. Results

### 3.1. Urine pH by General Characteristics

Table 1 shows the UpH by the general characteristics of the participants. The frequency of UpH < 5.5 was higher in females than in males (*p* = 0.126) and increased with age (*p* < 0.05). It was higher among those with a middle household income and those with a college degree or higher, but this was not statistically significant. It was higher among those without diabetes at 88.6% (*p* < 0.001), in the obese group at 37.5%, and in the non-smoking group at 80.5% (*p* < 0.001). It was as high as 90.4% in the group brushing teeth two times or more a day (*p* < 0.05), 66.1% in the group with CPI (<4 mm), and 44.4% in the group with 20–27 teeth (*p* < 0.001).

### 3.2. CPI by General Characteristics

Table 2 shows the periodontal tissue condition by the general characteristics of the participants. The CPI (≥4 mm) was higher in men and increased with age (*p* < 0.001). It was higher among those with a middle household income at 52.3% and high school graduates at 30.4%, but this was not statistically significant (*p* < 0.001).

It was higher among those without diabetes at 84.5%, in the obese group at 40.5% (*p* < 0.001), in the non-smoking group at 76.2%, and in the group brushing teeth two times or more a day at 88.1% (*p* < 0.001). It was 57.5% in the group with UpH ≥ 5.5 and 53.2% in the group with 20–27 remaining teeth (*p* < 0.001).

### 3.3. Number of Remaining Teeth by General Characteristics

Table 3 shows the number of remaining teeth by the general characteristics of the participants. The group with 0–19 remaining teeth had a higher frequency in women than men, with increasing age, and in a lower household income and education level (*p* < 0.001). It was higher among those without diabetes at 85.9% (*p* < 0.001) and in the obese group at 37.0% (*p* < 0.001). It was higher in the non-smoking and non-drinking groups at 81.2% (*p* = 0.132) and 60.6% (*p* < 0.001), respectively. It was as high as 80.2% in the group brushing teeth two times or more a day (*p* < 0.05), 58.8% in the group with UpH ≥ 5.5, and 53.3% in the group with CPI (<4 mm) (*p* < 0.001).

### 3.4. Association between UpH and CPI

Table 4 shows the results of the association between UpH and CPI. In Model 1, not adjusted for low UpH (<5.5), the odds ratio for CPI ≥4 mm was 1.27 times (95% CI 1.14–1.41). Model 2, adjusted for socioeconomic factors, showed an odds ratio for CPI ≥4 mm of 1.24 (95% CI 1.11–1.38), and Model 3, adjusted for socioeconomic and medical factors, showed an odds ratio for CPI ≥ 4 mm of 1.20 (95% CI 1.07–1.33). Finally, in Model 4, adjusted for socioeconomic, medical, and health-related behavioral factors, the odds ratio for CPI ≥ 4 mm was 1.19 (95% CI 1.06–1.33).

Model 1 unadjusted model. Model 2 adjusted for socioeconomic variables (sex, age, household income, and education). Model 3 adjusted for the same factors as model 2 plus general health variables (diabetes mellitus and BMI). Model 4 adjusted for the same factors as Model 3 plus health and behavior variables (smoking, alcohol drinking, and toothbrushing).

### 3.5. Association between Urine pH and the Number of Teeth

Table 5 shows the results of the association between UpH and the number of teeth. Model 1 for 0–19 teeth shows an odds ratio of tooth loss increase of 1.26 times in the UpH ≥ 5.5 group compared to the UpH < 5.5 group. In Model 2, adjusted for all variables, it increased 1.25 times (95% CI 1.06–1.48). Furthermore, in Model 1 for 20–27 teeth, the odds ratio increased 1.21 times. In Model 2, the odds ratio of tooth loss was 1.20 times (95% CI 1.07–1.34) in the UpH < 5.5 group.

Model 1 unadjusted model. Model 2 adjusted for socioeconomic variables (sex, age, household income, and education), general health variables (diabetes mellitus and BMI), and health and behavior variables (smoking, alcohol drinking, and toothbrushing).

## 4. Discussion

This study analyzed the association between UpH (<5.5), which may indicate systemic health status, CPI, and the number of remaining teeth using raw data from the 7^th^ KNHANES, representing the Korean adult population. A total of 12,689 adults aged 19 years and older who underwent periodontal examinations were examined. This is the first study to examine the association of UpH (<5.5) with oral indicators (CPI and the number of remaining teeth). Key findings from the study were as follows.

After adjusting for all variables, UpH (<5.5) was associated with CPI (≥4 mm) and fewer remaining teeth. For UpH (<5.5), the odds ratio for CPI (≥4 mm) was 1.19 times (95% CI: 1.06–1.33) while the risk of tooth loss was 1.25 times (95% CI: 1.06–1.48) for those with 0–19 remaining teeth and 1.20 times (95% CI: 1.07–1.34) for those with 20–27 teeth.

The results of this study show that there was no significant difference in the CPI by alcohol consumption (Table 2). The covariate of alcohol drinking is defined as the monthly alcohol intake. Non-drinkers were defined as individuals who had consumed less than one drink per month in the past year, while drinkers were those who had consumed one or more drinks per month in the past year. Therefore, it is presumed that such results were retrieved because the degree of alcohol consumption was not categorized in detail. Furthermore, regarding the number of remaining teeth according to alcohol consumption status, among the drinkers group, the percentage of individuals with 0–19 teeth was 39.4%, and the percentage with 28 teeth was 60.1% (Table 3). As mentioned earlier, this could be due to the issue of categorizing alcohol consumption, or alternatively, although not mentioned in the data, it is possible that non-drinkers exhibited similar results due to factors such as their older age and prevalence of systemic diseases.

This study also found that there was no significant difference in the number of remaining teeth by smoking status (Table 3). When considering the percentage distribution by the number of remaining teeth, the proportion of non-smokers was higher at 83.3% in the group with 28 teeth, while the proportion of non-smokers was lower at 81.2% in the group with 0–19 teeth. Looking at the results of CPI (≥4 mm) by the frequency of daily tooth brushing, the number of individuals with CPI (≥4 mm) was found to be higher in the group who brushed their teeth two or more times a day.

If we consider the cross-analysis in terms of the proportion of vertical sums, individuals who brushed their teeth two or more times a day accounted for 93.4% in the CPI (<4 mm) group, while they accounted for a lower proportion of 88.1% in the CPI (≥4 mm) group. Therefore, while theoretically valid when considering the proportions of the CPI (≥4 mm) and CPI (<4 mm) groups, it seems that further analysis is needed for individuals who brush their teeth two or fewer times a day.

In this study, UpH (<5.5) was associated with the Community Periodontal Index and number of remaining teeth. The causal relationship cannot be established at this point due to the inadequate literature on the association between UpH and oral health.

We considered insulin resistance as a major factor contributing to urine acidity [24,25].

Insulin resistance is the inability of the body’s cells and tissues to respond appropriately to normal levels of insulin. Causes include obesity, lack of exercise, advanced glycosylation end products (AGEs), excess free fatty acids (FFAs), stress, smoking, alcohol, or drugs [24,25]. It manifests in various outcomes, including a combination of metabolic disorders, lipotoxicity, glucotoxicity, and inflammation [26]. As such, it is associated with the pathophysiologic mechanisms of insulin resistance in the development of Mets, cardiovascular disease, and inflammation [14].

Previous studies reported that periodontitis causes colonization of bacteria such as *Aggregatibacter actinomycetemcomitans* and *Porphyromonas gingivalis* within the periodontal pocket, and that bacteria and antigens introduced into the bloodstream via local epithelial ulcers induce the release of inflammatory mediators. This triggers the inflammation cascade [27,28,29].

Furthermore, research suggests a bidirectional relationship between periodontitis and insulin resistance, where chronic systemic inflammation exacerbates periodontitis by inducing the expression of tumor necrosis factor-α in periodontal tissues [30]. Such exacerbation can contribute to tooth loss by promoting the resorption of periodontal tissues.

Since this study did not analyze the data based on the measurements of insulin resistance, future research should be conducted for investigation in relation to insulin resistance and type 2 diabetes.

The findings of this study were similar to those of cross-sectional studies on the association between Mets and decreased masticatory capacity. Participants with poor masticatory function, categorized based on the total number of functioning teeth and masticatory capacity, had a more than 2.5-fold increased risk for diseases such as Mets (diabetes, hypertension, abdominal circumference, and hypercholesterolemia) [31]. Similarly, metabolic syndrome (MetS) and periodontitis exhibit a bidirectional relationship, arising from factors related to immune and inflammatory responses [32].

The mechanism linking periodontitis and MetS involves the infiltration of proinflammatory cytokines from the gingiva into the bloodstream, leading to increased oxidative stress, triggering insulin resistance and atherosclerotic changes, potentially resulting in the development of MetS [33].

Previous studies have noted that risk factors associated with UpH, such as insulin resistance and MetS, can also be risk factors for oral diseases. This bidirectional relationship partially explains the findings of this study.

This study has several strengths. This is the first study to analyze the association between UpH, a readily measurable urinary parameter, and oral health (CPI and number of remaining teeth). A representative raw dataset from the Korea National Health and Nutrition Examination Survey was used.

This study also has the following limitations. First, as it was a cross-sectional study, causal relationships could not be confirmed. Second, the number of remaining teeth applied in this study may have varying definitions depending on the status of the subjects. For example, the number of remaining teeth in individuals with poor oral health may be inappropriate to reflect a healthy lifestyle. Therefore, in this study, the periodontal status was also considered to complement the limitations of the number of remaining teeth. Subsequently, it would be necessary to consider data that allows for a more specific evaluation of the oral health status. Third, although UpH is associated with specific conditions and medications, this aspect was not considered in this study. Therefore, bias may occur as a result. Hence, future research designs need to consider specific conditions and medication interactions related to UpH.

This study investigated the association between UpH, a readily measurable urinary parameter, and oral health (CPI and number of remaining teeth). The findings of this study suggest that UpH could serve as a valuable tool for predicting and screening for oral diseases in both clinical and community settings following further systematic research. Additionally, this parameter holds the potential to contribute to preventive health promotion programs aimed at addressing non-communicable diseases. In the research domain, UpH could be a useful biomarker in various biomarker research, including clinical, longitudinal, and cohort studies.

## 5. Conclusions

Based on the results of this study, UpH (<5.5) was associated with CPI and the number of remaining teeth. Since UpH changes with Mets, insulin resistance, and a healthy lifestyle, comprehensive healthcare is needed for systemic and oral health.

## Figures and Tables

**Table 1:** Characteristics of the study population stratified by urine pH.

Characteristics		Urine pH				*p*-Value **
		<5.5		≥5.5		
		n	% *	n	% *	
Sex (n = 12,643)	Man	2222	44.0	3413	42.5	0.126
	Woman	2680	56.0	4328	57.5	
Age (n = 12,643)	19–29	493	10.8	977	13.2	0.005
	30–39	747	14.6	1240	15.8	
	40–49	964	18.7	1354	17.0	
	50–59	1009	21.4	1429	19.5	
	≥60	1689	34.4	2741	34.5	
House income (n = 12,606)	Lower	1000	19.9	1482	18.9	0.575
	Median	2500	50.7	4037	52.1	
	Upper	1390	29.4	2197	29.0	
Education	≤Primary school	993	20.9	1523	19.3	0.291
(n = 12,106)	Middle school	485	10.4	720	10.1	
	High school	1443	31.5	2431	33.3	
	≥College	1757	37.2	2754	37.3	
Diabetes (n = 12,643)	Absence	4302	88.6	7098	91.9	<0.001
	Presence	600	11.4	643	8.1	
BMI	Underweight	167	3.5	312	4.2	<0.001
(n = 12,347)	Normal	1716	36.8	2974	40.2	
	Overweight	1059	22.2	1742	23.0	
	Obesity	1850	37.5	2527	32.6	
Smoking (n = 12,543)	Non-smoker	3891	80.5	6393	83.7	<0.001
	Smoker	977	19.5	1282	16.3	
Alcohol drinking (n = 12,549)	Non-drinker	2254	46.5	3568	46.3	0.806
	Drinker	2615	53.5	4112	53.7	
Tooth brushing/day	≤1	482	9.6	652	8.2	0.034
(n = 12,274)	≥2	4274	90.4	6866	91.8	
CPI	<4 mm	3092	66.1	5247	71.3	<0.001
(n = 12,178)	≥4 mm	1608	33.9	2231	28.7	
Number of teeth	0–19	831	17.1	1218	15.3	<0.001
(n = 12,643)	20–27	2170	44.4	3237	41.2	
	28	1901	38.5	3286	43.5	

* Weighted %; ** *p*-Value was calculated by complex sample chi-square test.

**Table 2:** Characteristics of the study population stratified by CPI.

Characteristics		CPI				*p*-Value **
		<4 mm		≥4 mm		
		n	% *	n	% *	
Sex (n = 12,689)	Man	3417	37.8	2128	52.1	<0.001
	Woman	5291	62.2	1853	47.9	
Age (n = 12,689)	19–29	1501	17.8	61	1.7	<0.001
	30–39	1806	20.0	294	7.1	
	40–49	1768	19.8	647	15.2	
	50–59	1466	17.8	1005	26.4	
	≥60	2167	24.5	1974	49.6	
House income (n = 12,656)	Lower	1331	15.1	1009	25.0	<0.001
	Median	4563	51.9	2069	52.3	
	Upper	2796	33.0	888	22.7	
Education	≤Primary school	1172	13.4	1147	29.8	<0.001
(n = 12,110)	Middle school	654	7.8	522	14.7	
	High school	2827	34.7	1134	30.4	
	≥College	3686	44.0	968	25.1	
Diabetes (n = 12,689)	Absence	8134	93.7	3358	84.5	<0.001
	Presence	574	6.3	623	15.5	
BMI	Underweight	379	4.5	96	2.3	<0.001
(n = 12,390)	Normal	3523	42.2	1210	32.0	
	Overweight	1839	21.4	955	25.2	
	Obesity	2760	31.8	1628	40.5	
Smoking (n = 12,571)	Non-smoker	7344	85.4	2966	76.2	<0.001
	Smoker	1292	14.6	969	23.8	
Alcohol drinking (n = 12,580)	Non-drinker	3921	45.3	1830	46.8	0.216
	Drinker	4716	54.7	2113	53.2	
Tooth brushing/day	≤1	611	6.6	470	11.9	<0.001
(n = 12,400)	≥2	7934	93.4	3385	88.1	
UpH	<5.5	3092	36.8	1608	42.5	<0.001
(n = 12,178)	≥5.5	5247	63.2	2231	57.5	
Number of teeth	0–19	894	10.0	781	20.0	<0.001
(n = 12,689)	20–27	3469	39.7	2123	53.2	
	28	4345	50.3	1077	26.9	

* Weighted %; ** *p*-Value was calculated by complex sample chi-square test.

**Table 3:** Characteristics of the study population stratified by number of teeth.

Characteristics		Number of Teeth						*p*-Value **
		0–19		20–27		28		
		n	% *	n	% *	n	% *	
Sex (n = 13,199)	Man	1029	47.3	2373	40.3	2391	42.7	<0.001
	Woman	1151	52.7	3223	59.7	3032	57.3	
Age (n = 13,199)	19–29	2	0.1	393	7.4	1167	22.3	<0.001
	30–39	11	0.6	745	13.0	1346	23.9	
	40–49	47	2.1	951	16.5	1421	25.1	
	50–59	226	10.8	1358	25.5	909	17.7	
	≥60	1894	86.4	2149	37.6	580	11.1	
House income (n = 13,161)	Lower	1079	47.7	1086	19.2	476	9.1	<0.001
	Median	881	42.1	2945	52.1	2985	54.4	
	Upper	209	10.1	1547	28.7	1953	36.5	
Education	≤Primary school	1175	56.0	1136	20.3	318	6.5	<0.001
(n = 12,566)	Middle school	300	15.3	680	13.0	253	5.1	
	High school	371	20.0	1768	33.9	1889	36.4	
	≥College	166	8.7	1750	32.8	2760	52.0	
Diabetes (n = 13,199)	Absence	2988	85.9	5493	90.2	6077	96.7	<0.001
	Presence	488	14.1	619	9.8	200	3.3	
BMI	Underweight	74	3.8	192	3.5	234	4.3	<0.001
(n = 12,880)	Normal	714	34.4	2012	38.4	2195	41.5	
	Overweight	539	24.8	1247	22.8	1114	21.3	
	Obesity	772	37.0	2008	35.3	1779	32.8	
Smoking (n = 13,061)	Non-smoker	1746	81.2	4520	82.2	4448	83.3	0.132
	Smoker	381	18.8	1027	17.8	939	16.7	
Alcohol drinking (n = 13,069)	Non-drinker	1308	60.6	2661	47.7	2113	39.9	<0.001
	Drinker	823	39.4	2890	52.3	3274	60.1	
Tooth brushing/day	≤1	392	19.8	483	8.2	317	5.5	<0.001
(n = 12,759)	≥2	1525	80.2	5006	91.8	5036	94.5	
Urine pH	<5.5	874	41.2	2350	40.4	2196	35.7	<0.001
(n = 14,024)	≥5.5	1294	58.8	3519	59.6	3791	64.3	
CPI	<4 mm	894	53.3	3469	62.9	4345	81.0	<0.001
(n = 12,689)	≥4 mm	781	46.7	2123	37.1	1077	19.0	

* Weighted %; ** *p*-Value was calculated by complex sample chi-square test.

**Table 4:** Multivariable association between UpH and CPI ≥ 4 mm.

	Model 1	Model 2	Model 3	Model 4
UpH	Unadjusted	Adjusted Odds Ratio (95% Confidence Interval)		
(1) <5.5	1.27 (1.14–1.41) **	1.24 (1.11–1.38) **	1.20 (1.07–1.33) *	1.19 (1.06–1.33) *
(2) ≥5.5	Reference	Reference	Reference	Reference

Data are presented as OR (95% CI). OR: odds ratio; CI: confidence interval, * *p* < 0.01 and ** *p* < 0.001.

**Table 5:** Association between exposure variable number of teeth 0–19 and 20–27.

	28 versus 0–19		28 versus 20–27	
	Model 1	Model 2	Model 1	Model 2
UpH	Unadjusted	Adjusted OR (95% CI)	Unadjusted	Adjusted OR (95% CI)
(1) <5.5	1.26 (1.09–1.45) *	1.25 (1.06–1.48) *	1.21 (1.10–1.33) **	1.20 (1.07–1.34) *
(2) ≥5.5	Reference	Reference	Reference	Reference

Data are presented as OR (95% CI). OR: odds ratio; CI: confidence interval, * *p* < 0.01 and ** *p* < 0.001.

## Data Availability

The data from the KNHANES VII survey can be accessed and downloaded from the KNHANES homepage (URL: https://knhanes.kdca.go.kr/knhanes/eng/index.do accessed on 15 January 2024).
